# What is the Most Important Leg and Discipline in Triathlon Mixed Team Relays?

**DOI:** 10.5114/jhk/167088

**Published:** 2023-07-06

**Authors:** Jesús Martínez-Sobrino, Santiago Veiga, Jesús S. del Cerro, José María González-Ravé

**Affiliations:** 1 Departamento de Deportes, Universidad Politécnica de Madrid, Madrid, Spain.; 2 Spanish Triathlon Federation, Madrid, Spain.; 3 Department of Statistics, University of Castilla-La Mancha, Toledo, Spain.; 4 Sports Training Laboratory, Faculty of Sports Sciences, University of Castilla-La Mancha, Toledo, Spain.

**Keywords:** athletic performance, swimming, cycling, running, competition

## Abstract

This study aimed to determine the importance of the different relay legs and sport disciplines in the overall result of the triathlon Mixed Team Relay (MTR) events. The study analysed the results of 80 Mixed Team Relay triathlon teams (n = 320 professional triathletes) corresponding to the top ten finishers at the World Championships in Hamburg from 2013 to 2020. Split times, average speeds, time behind the race leader (gap), partial and finishing positions, as well as the rank positions of every segment, relay leg, and overall race were computed. Decision tree analyses were conducted as a predictive method for the overall results, and correspondence analyses were conducted to examine the relationship between the different relay legs and segments and the finishing positions. Running was the variable with the greatest importance (32%) in the overall result, followed by female team members (17%) and the third relay leg (17%). The swimming segments (1%) and the fourth relay leg (1%) had the lowest relevance. Medallist relay teams were characterised by cycling and running faster than 10.99 m/s and 5.59 m/s, respectively, with time gaps of less than 43 seconds by the end of the third relay leg. A reliable and accurate prediction model for the medallists' and finalists' team positions in the Mixed Team Relay triathlon was obtained. The running disciplines and performance of female team members, especially in the third leg, were ascertained to be the most significant determinants for the overall Mixed Team Relay result.

## Introduction

In 2009, the International Triathlon Union (ITU) announced the first Mixed Team Relay (MTR) World Championships, held in Des Moines (Iowa, USA); 12 years later, the MTR triathlon made its Olympic debut at the 2021 Tokyo Olympic Games. In this discipline, teams are composed of two male and two female triathletes and each of them must complete a super-sprint triathlon (between a 250–300 m swim, a 5–8 km bike, and a 1.5–2 km run) before relaying the next leg to a teammate. Until 2022, the order of participants within each team was female-male-female-male, but since then the format has been changed to male-female-female-male (World Triathlon Competition Rules, 2022). Interestingly, all triathlon MTR World Championships between 2013 and 2020 were hosted in Hamburg (Germany) in a similar circuit and under similar race conditions.

There is a lack of research on the performance determinants in MTR events. To the best of our knowledge, one recent article examined the characteristic features of successful relay teams (Quagliarotti et al., 2022) and it was concluded that cycling was the most decisive discipline in the MTR, while the third relay leg was the most important in predicting the overall relay time. Performance determinants have been extensively studied in individual triathlon races (Cuba-Dorado et al., 2020; Gadelha et al., 2020; Ofoghi et al., 2016; Olaya et al., 2021; Sousa et al., 2021; Vleck et al., 2006, 2008) and it was observed that swimming was the best predictor for overall performance in the Olympic distance triathlon (Sousa et al., 2021). In addition, finishing the swim segment in the first pack (Landers et al., 2008; Peeling and Landers, 2009) and achieving a split time closer to the fastest swimming time (Ofoghi et al., 2016) were also critical variables. However, there is a lack of consensus between researchers, as some other studies identified the running segment as the most decisive discipline (Gadelha et al., 2020; Ofoghi et al., 2016; Piacentini et al., 2019; Vleck et al., 2006, 2008). The importance of different race segments (swimming, cycling, and running) in overall performance also seems to depend on the triathlon distance (Sousa et al., 2021). For example, previous studies in sprint distance triathlons highlighted the cycling segment as the most important for final placing (Olaya et al., 2021; Sousa et al., 2021). On the other hand, the transition parts between the swimming, cycling, and running segments showed poor correlation with overall performance in the sprint (Olaya et al., 2021) and Olympic distance (Cejuela et al., 2012, 2013), as they represent a small percentage of the race time.

Regarding race tactics, successful male and female elite triathletes were reported to complete each race component very close to the quickest rank time, showing no weaknesses (Ofoghi et al., 2016). Furthermore, Vleck et al. (2006) found that the highest positioned triathletes swam faster during the first 400–500 m, cycled significantly slower due to not having to bridge the gap, and ran faster than their competitors. During transition phases, minimising time loss to leaders seemed to play a major role in ranking higher at the end of the race (Cejuela et al., 2013; Millet and Vleck, 2000; Piacentini et al., 2019). Another important aspect for race tactics also present in MTR races is the drafting effect between competitors. Research reported a reduction in oxygen consumption, lactate concentration, metabolic expenditure, and the rate of perceived exertion (RPE) for those who exhibited the drafting effect during swimming (Chatard and Wilson, 2003; Janssen et al., 2009), cycling (Blocken et al., 2018; McCole et al., 1990), and running (Beaumont et al., 2021; Schickhofer and Hanson, 2021). The drafting effect is determined by the type of the pack formation, triathlete placement, distance from rivals, and speed (Blocken et al., 2018; Puce et al., 2022; Schickhofer and Hanson, 2021), thus affecting the overall MTR performance.

Additionally, there seems to exist a specific gender effect when examining the determinants of triathlon performances. For example, the bike discipline was shown to be more important for overall performance in female than in male elite triathletes (Cejuela et al., 2012; Piacentini et al., 2019; Vleck et al., 2008). Lower speeds, smaller bike packs, more heterogeneous levels, difficulties in “catching up”, and the fact that female triathletes are more affected by changes in the slope may explain the higher importance attached to them having a good cycling level for success compared to their male counterparts (Le Meur et al., 2009; Piacentini et al., 2019; Vleck et al., 2008). In other sport disciplines, it was also observed that women’s relay legs played a critical role in overall team performance, for example in swimming mixed relay events (Veiga et al., 2021).

Therefore, the aim of the present research was to determine the importance of different relay legs and disciplines on the final performance in MTR triathlon events, as a function of a team’s competitive level. It was hypothesised that performance during female athletes’ relay legs would have a greater influence on the overall team performance, with special focus attached to the cycling segment.

## Methods

### Participants

The results of 80 teams corresponding to the top ten finishers at the MTR World Championships held in Hamburg between 2013 and 2020 were analysed. This represented a total of 160 female and 160 male world elite triathletes (n = 320) from 19 different countries. The race venue for all selected races was in the same city and with the same race distances (300 m, 6.5 to 7 km, and 1.7 km for the swimming, cycling, and running disciplines, respectively), thus facilitating a more reliable analysis of all the data in highly similar scenarios. The time spent transitioning between the swimming, cycling, and running segments was not included in the analysis.

### Measures

The data collection was performed through the World Triathlon database in its public domain at www.triathlon.org; this included the split times (in seconds) of each discipline

(swimming, cycling, and running), the split time of each relay leg (first to fourth), and the overall relay team time. The average speeds (m/s) were also calculated; to aid interpretation, paces were expressed as time per 100 m (s) for swimming and in m/s for cycling and running. Partial and finishing positions of relay teams as well as rank positions for each team within each relay leg and discipline were also computed.

### Statistical analysis

Statistical analyses were performed using R software (version 4.1.2 for Windows). Speed, time behind the race leader (gap), intermediate and final positions, as well as the rank positions were compared between medallists (top three finishers) and finalists (fourth to tenth finishing positions). Comparisons were completed by relay legs (leg 1, 2, 3 or 4), disciplines (swimming, cycling, and running) and gender (male or female). Decision tree analyses were conducted to provide a graphical representation that visually indicated the potential outcomes, costs, and consequences of the interaction of different variables on the relay performance. This technique is used in machine learning as a classification method, and in this work it allowed us to predict whether a team could reach a medallist overall position in the MTR. A receiver operating characteristic (ROC) curve was performed to analyse the predictive capacity of the model.

In addition, a correspondence analysis was performed to evaluate the relationships between qualitative variables through the structure of their joint frequencies, i.e., according to the type of interaction between these variables. In our case, we studied the relationship between the final position of the relay teams and their rank position in different disciplines and relay legs. Therefore, 16 models were computed, which were subsequently reduced to two dimensions and graphically represented in ℝ^2^. The goodness-of-fit measures should be interpreted as the percentage of original information that was retained in the process of dimension reduction through this technique.

## Results

In the estimation of the classification tree, one output was the relative importance of each of the predictor or explanatory variables, with running being the variable with the greatest weight (32%), followed by the performance of female team members (17%), and performance during the third relay leg (17%). Moreover, performance during the swimming segments (1%) and in the fourth relay leg (1%) showed the lowest importance in the overall results.

**Figure 1:**
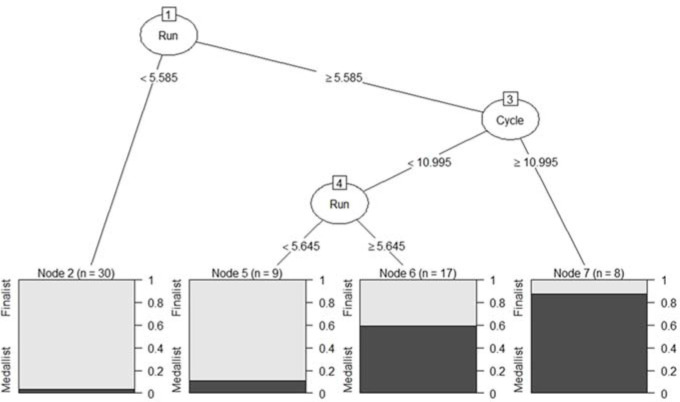
Decision tree for the classification of finalist and medallist teams based on the speed (m/s) of the cycling and running segments at the triathlon Mixed Team Relay World Championships from 2013 to 2020.

**Figure 2:**
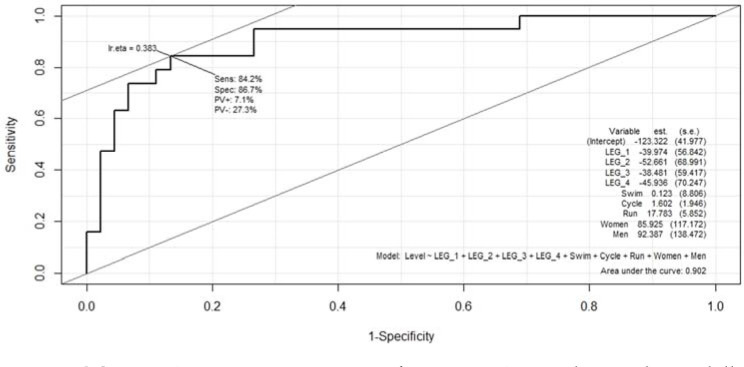
ROC curve (sensitivity: y-axis; specificity: x-axis) according to the medallist and finalist positions at the triathlon Mixed Team Relay World Championships from 2013 to 2020. *AUC = area under the curve*.

**Figure 3:**
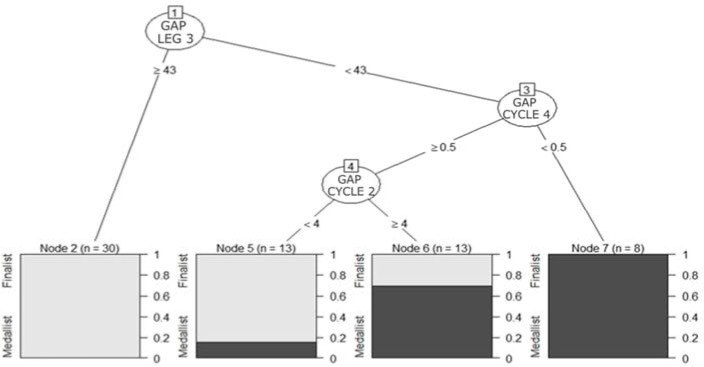
Decision tree for the classification of finalist and medallist teams based on the time gap(s) behind the leader at the triathlon Mixed Team Relay World Championships from 2013 to 2020. *Note. Leg 3 responds to the existing time gap(s) at the end of the third leg, and cycle 2 or cycle 4 to the end of the bike segment in the second and fourth legs, respectively*.

Medallist relay teams were characterised (Figure 1) by displaying a running speed higher than 5.59 m/s and a cycling speed faster than 10.99 m/s (node 7). In case teams did not achieve the mentioned cycling velocity, their running velocity should be higher than 5.65 m/s to obtain a medallist finishing position (node 6). Once the model was estimated from the validation sample, the accuracy was 81.25% and the area under the curve (AUC) was 0.902 (Figure 2), indicating the model’s high predictive capability. In addition, the sensitivity of the model in predicting medallists and finalists was 100% and 72.7%, respectively. The results of the classification tree (Figure 3) also illustrated that teams with gap times greater than 43 s from race leaders at the end of the third relay leg could not achieve a medallist position (node 2). In cases in which the gap time was less than 43 s and the subsequent gap time at the cycling segment of relay leg four was less than 0.5 s, teams could still achieve a medal position (node 7).

**Figure 4:**
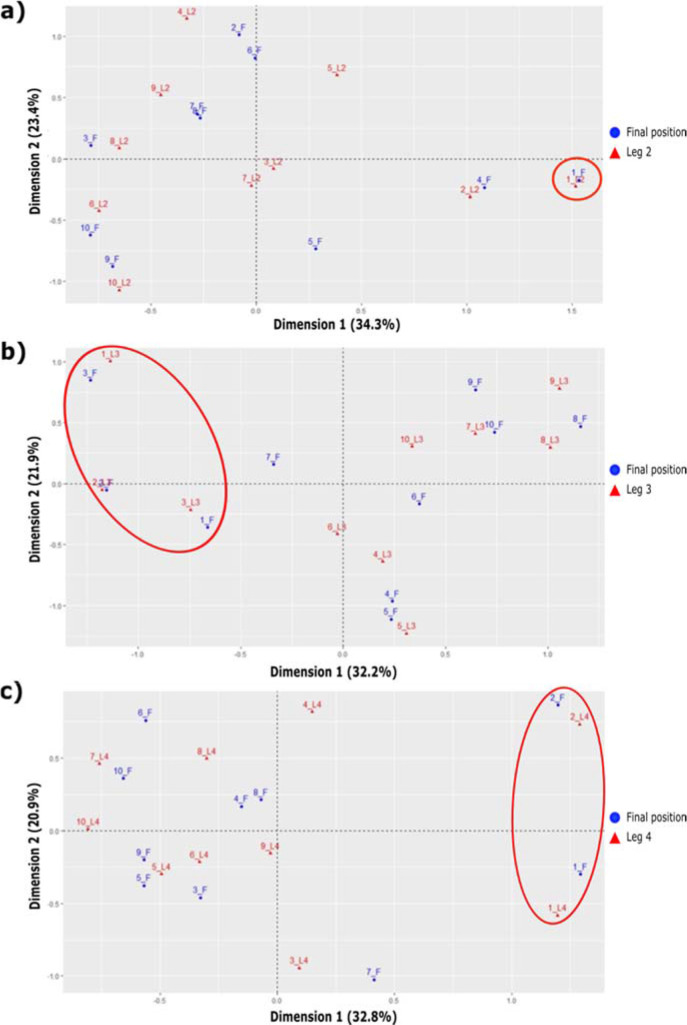
Correspondence analysis between the final position (F) and the rank in relay a) leg 1 (L1), b) leg 2 (L2), and c) leg 3 (L3).

**Figure 5:**
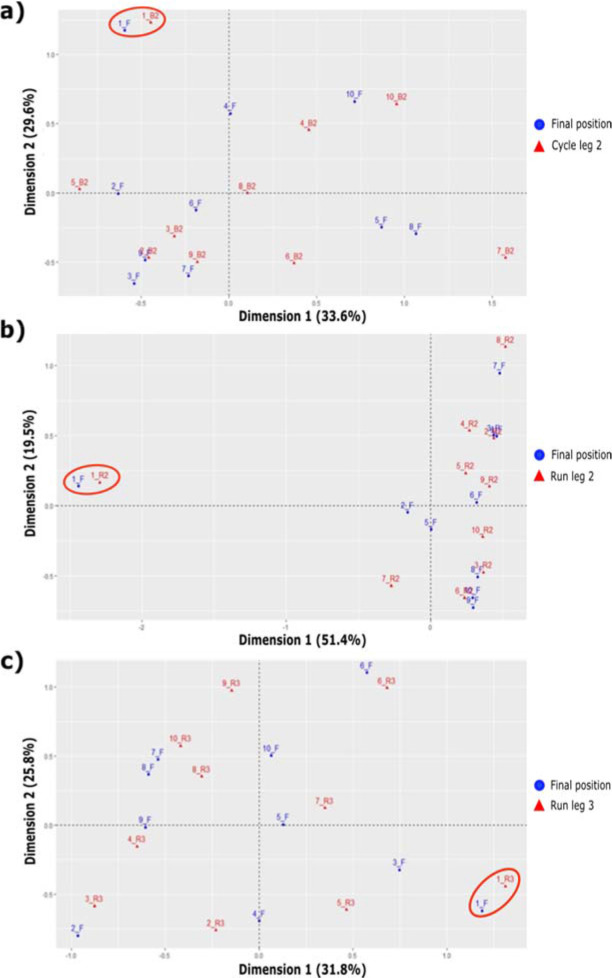
Correspondence analysis between the final position (F) and the rank in a) cycle leg 2 (B2), b) run leg 2 (R2), and c) run leg 3 (R3).

The correspondence analysis revealed a very high association between the winning relay teams (first finishing position) and achieving the best time split in the second relay leg. An association between achieving a medallist position and obtaining one of the three best times in the third relay leg, and between finishing in the first or the second position and displaying the best or second-best time in the fourth relay leg was also observed (Figure 4). No associations were found between the finishing position and the rank in the swim split in any of the four relay legs. However, a strong association was identified in the winning teams achieving the fastest rank in the cycling split of the second relay leg and the fastest rank in running segments, especially in the second and third relay legs (Figure 5). In all models, the goodness-of-fit ranged between 50% and 70%, which is significant. The relationships between partial and finishing positions increased until the beginning of the third swimming relay leg. From then on, no further increases in the relationships between partial and finishing positions were detected until the third running leg, where relationships increased to the end of the race.

## Discussion

The present study aimed to determine the importance of different relay legs and sports disciplines in teams’ overall performance during the triathlon MTR World Championships. Despite the MTR discipline being part of the Olympic programme since 2021, there has been a lack of research exploring race performance determinants. Data from the last eight World Championships held in Hamburg (2013 to 2020) under similar race conditions revealed that running, followed by the cycling segment, was the most important discipline for the overall MTR performance. In terms of the relay legs, those carried out by women triathletes (first and third) presented greater weight in the overall result, with the third leg being the most relevant.

Running performance was the best predictor of achieving a medallist team position in the MTR (32%) and its strong association with the winning team positions was especially relevant in the second and third relay legs. This was in line with data from the Olympic distance triathlon where most male and female medallists displayed running split times within 10 s of the best ranking or within the five best ranking positions in the running segment (Ofoghi et al., 2016). However, these data are in contrast with the analysis of all team participants during the MTR World Championships from 2014 to 2019 (Quagliarotti et al., 2022), where regression linear models indicated that cycling was the most important segment and running was the least important sport discipline in medallist teams. Indeed, the importance of running segments in triathlon performance would be expected to decrease with triathlon events over shorter distances like in MTR (Sousa et al., 2021). A key aspect could be the lower benefit from drafting strategies in the running segment regarding other disciplines (Olaya et al., 2021). Our results reported that to achieve a medallist position, running velocities should be faster than 5.59 m/s (≤ 2:59 min/km), or faster than 5.65 m/s (≤ 2:57 min/km) in cases in which the cycling segment was slower than 10.99 m/s (< 39.5 km/h). These velocities refer to the specific constraints of the Hamburg race venues but, compared to previous data, are considerably faster than ~3:25 and ~3:49 min/km required for men and women, respectively, to achieve one of the top three places in the Olympic distance triathlon (Gadelha et al., 2020).

For cycling segments, a high association was detected with relay performance (12%), specifically between the winning team positions and performance in the cycling segment of the second relay leg (male athlete). This could be related to the higher time proportion of cycling relative to the total race (Quagliarotti et al., 2022; Sousa et al., 2021) and to the strategic component related to the formation of small packs between competitors. Indeed, triathletes grouped in leading packs would have a critical advantage compared to triathletes with rear partial positions and gap times with the leaders (Blocken et al., 2018). Due to the higher speeds accomplished during cycling, the benefits of drafting in the leading packs could be critical in achieving faster average velocities during this segment (McCole et al., 1990) compared to other segments such as running. The fact that the first relay leg begins with all participants being grouped likely determines the occurrence of most athletes moving in the main pack during the first bike segment. However, at the beginning of the second cycling leg, the main group is much more fractionated in small packs, and this can represent a window of opportunity for teams with a fast male athlete. In the remaining relay legs (3 and 4), the distances with respect to the other competitors may already be large and difficult to recover from, which would explain their lower influence on the finishing positions. Pacing data indicated that cycling velocities at or higher than 10.99 m/s (≥ 39.5 km/h) would vastly increase teams’ medal chances if the run is performed close to the 2:59 min/km pace. This would mean riding faster than the ~39.5 and ~33.5 km/h reported in Olympic distance triathlon competitions for male and female medallists, respectively (Gadelha et al., 2020).

As for swimming, our analysis showed low importance of this segment in the overall result (1%). This may be due to the smaller temporal proportion (~18%) of this segment with respect to the total (Quagliarotti et al., 2022) and to the small time differences that can be marked in 300 m swimming. Previous findings in Olympic distance triathlons reported that swimming performance did not discriminate between the overall positions among the top 16 in both men and women (Piacentini et al., 2019).

Assessing the weight of the relay legs in the MTR results, female relay legs (1 and 3) appeared to have a greater influence on the overall result (17%) than male relay legs (2 and 4) (8%). This could be explained by a greater relative time of female versus male relay legs (≈26% vs. ≈24%), but also by the greater difficulty of females bridging the gaps during solo or group riding (Piacentini et al., 2019). Specifically, performance in the third relay leg (female athlete) reported the highest association with the medallist team positions and accounted for 17% of overall relay performance. This is consistent with Quagliarotti et al.'s (2022) findings regarding MTR and could be explained by the greater pack fragmentation of the race compared to the first relay leg (also a female relay leg). Similarly, the second relay leg seemed to have some weight within the overall result (8%) and a high relationship with the winning position for the best split time. On the other hand, the first and the fourth relay legs were found to be the least relevant for overall performance (accounting for 3% and 1%, respectively), but the fourth leg showed a high association with the winning position. For this reason, the decision tree analysis revealed that no time gap should exist between the teams at the beginning of the last running segment; it also highlighted that the third relay leg should be finished within 43 s from the leader to keep medal chances. Therefore, the win would depend on the last relay leg performance, particularly during the running segment.

The results of this study suggest that the most important decision for coaches and national selectors in MTR teams is the selection and performance of the female triathletes. In addition, successful (in terms of medals) teams should be composed of four great runners and should place their best male and female triathlete in the second and third relay legs. Among those teams competing for the first position, the fastest male runner should be placed in the last leg to aim for the best split time. All these recommendations should be interpreted in relation to the different race scenarios and precautions should be taken when applying these results to other competitions. For example, environmental factors (e.g., wind, air temperature, water temperature, humidity) could have influenced such analysis over several years.

## Conclusions

A reliable and accurate prediction model for the medallists' and finalists' team positions was obtained from triathlon MTR World Championships data spanning from 2013 to 2020. The running discipline and the performance of female triathletes, especially in the third relay leg, were the most significant variables in determining the overall relay performance. On the contrary, the swimming segment and the fourth relay leg appeared to be the variables with the least influence on the MTR result. However, when it comes to elucidating the winning position, performance in the fourth leg could be critical. These results can provide coaches and national performance directors with vital information about the relay team composition and the profile of the selected triathletes.
